# The expression, regulation, and function of human endogenous retroviruses in genitourinary cancers

**DOI:** 10.1038/s41420-025-02820-2

**Published:** 2025-11-28

**Authors:** Wenjie Ma, Chencheng Ji, Abudukelimu Abudushataer, Ning Liu, Tao Xu, Kunlun Zhao, Yiguan Qian, Paerhati Tuerxun, Xiaotian Jiang, Zhongli Xiong, Min Wang, Ruipeng Jia, Zheng Xu, Yang Li, Yu-Zheng Ge

**Affiliations:** 1 Department of Urology, Nanjing First Hospital, Nanjing Medical University, Nanjing, Jiangsu PR China; 2 Department of Urology, Yining People’s Hospital, Yining, Xinjiang PR China; 3 Department of Urology, People’s Hospital Campus of Yining General Hospital, Yining, Xinjiang PR China; 4 General Clinical Research Center, Nanjing First Hospital, Nanjing Medical University, Nanjing, Jiangsu PR China

**Keywords:** Urological cancer, Tumour virus infections, Cancer genomics

## Abstract

Human endogenous retroviruses (HERVs), constituting approximately 8% of the human genome, represent genomic remnants of ancestral retroviral infections that colonized the germline through evolutionary processes. While most HERVs remain epigenetically silenced, their reactivation through environmental stimuli or epigenetic dysregulation enables participation in oncogenesis via viral mimicry, immunomodulation, and insertional mutagenesis. Substantial evidence now implicates aberrant HERVs activity across urologic malignancies—including prostate cancer, renal cell carcinoma (RCC), bladder cancer, and testicular germ cell tumors—where cancer-type-specific mechanisms drive tumor development and progression. These encompass androgen-responsive HERV-K activation in prostate malignancies, hypoxia-inducible factor-mediated ERV immunogenicity in RCC, HERV-derived microRNA silencing of tumor suppressors in bladder cancer, and DNA hypomethylation-associated HERV expression in testicular germ cell tumors. This review synthesizes fundamental HERV biology with recent advances in their diagnostic and therapeutic applications for urologic neoplasms. Key clinical translations include ERV-based stratification models predicting immune checkpoint inhibitor response in metastatic RCC, HERV-E-targeted adoptive T cell therapies, and noncoding RNA biomarkers for early bladder cancer detection. We further discuss unresolved mechanistic paradoxes such as contradictory prognostic associations between HERV superfamily expression and PBRM1 inactivation in RCC, concluding with priorities for future research: validating HERV-derived neoantigens in immunotherapy platforms, optimizing epigenetic priming strategies to enhance viral mimicry effects, and establishing standardized HERV signatures as clinical biomarkers through multi-institutional cohorts.

## Facts

- HERVs reactivation is epigenetically regulated: DNA hypomethylation and histone modifications derepress HERVs loci in prostate, renal, bladder, and testicular cancers, driving oncogenic transcription.
- Epigenetic dysregulation is the primary trigger for aberrant HERVs expression, enabling viral mimicry, oncogene activation, and immune evasion across genitourinary malignancies.
- HERVs–based biomarkers enable early detection and prognosis prediction.

## Open questions

- Can HERVs-derived neoantigens be robustly targeted in clinical immunotherapy platforms without off–tumor toxicity?
- How can multi-institutional cohorts establish consensus HERVs expression signatures as diagnostic tools across heterogeneous urologic malignancies?
- Which epigenetic priming strategies optimally enhance HERVs-induced interferon responses in “cold” genitourinary tumors?

## Introduction

**Fig. 1:**
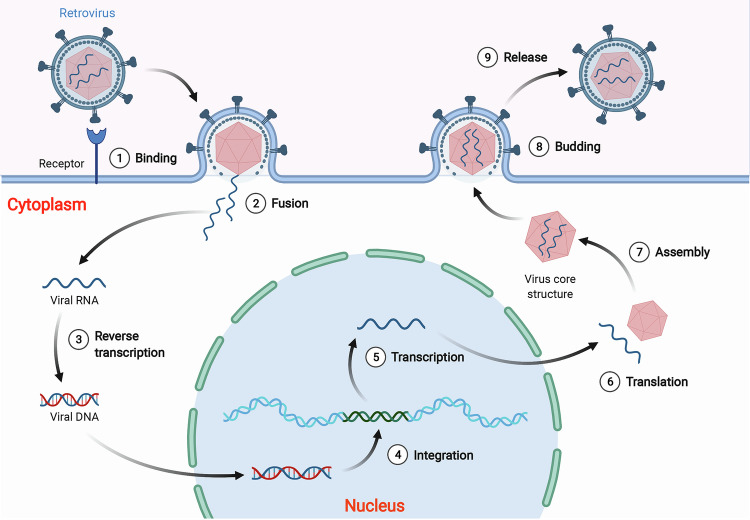
The life cycle of retroviruses. Retroviral infection is a complex and multistep process that involves several distinct stages. Initially, the retrovirus attaches to the cell surface receptor and delivers its viral core to the cytoplasm (steps 1 and 2). Upon entry into the target cell, the viral RNA undergoes reverse transcription, which generates double-stranded DNA that can integrate into the host genome as a provirus (steps 3 and 4). Following integration, various proviruses are transcribed according to their distinct transcriptional start sites, and the resulting transcripts are translated and assembled in the cytoplasm (steps 5, 6 and 7). Finally, the assembled viral particles are bound to the cell membrane and released (steps 8 and 9). (Created with BioRender.com).

Human endogenous retroviruses (HERVs) represent remnants of ancestral exogenous retroviral infections that became integrated into the germline, comprising approximately 8% of the human genome [1]. Retroviral particles carry genomic RNA, which undergoes reverse transcription into double-stranded DNA upon entering a target cell; this DNA subsequently integrates into the host genome. The integrated viral sequence, termed a provirus, contains regulatory elements (promoters, enhancers) and genes encoding structural proteins and enzymes [2]. For productive infection, the provirus is transcribed and translated, followed by assembly and release of new viral particles [3–7] (Fig. 1). While retroviruses typically infect somatic cells, occasional germline infection results in the vertical inheritance of the provirus as part of the host chromosome. This process, mediated by natural selection and genetic drift, leads to the formation of endogenous retroviruses (ERVs) [2, 8, 9].

Genitourinary cancers impose a significant global health burden. The GLOBOCAN 2020 report estimated 7.66 million new cases of urologic cancer worldwide, with over 780,000 associated deaths. Among these, prostate cancer is the second most frequently diagnosed malignancy in men (14.1% of male cancer cases) and the fifth leading cause of male cancer mortality globally (6.8% of deaths). Both bladder and kidney cancers rank among the ten most common cancers in both sexes [10]. Although diagnostic and therapeutic advancements have improved patient prognosis [10–12], the disease burden is projected to rise [13, 14]. Consequently, further exploration of oncogenic mechanisms and the development of innovative diagnostic and therapeutic strategies are imperative to enhance clinical outcomes for patients with urological cancers.

Previous researchers investigated HERVs as genomic parasites because many HERVs have lost the capacity to form functional viral particles owing to mutations and deletions of coding genes. Nevertheless, more recent studies have shown that HERVs participate in various biological processes in humans and are associated with dysregulation in numerous diseases, particularly malignant tumors [15, 16]. Nonetheless, the study of HERV expression and function in urologic neoplasms remains an area requiring further study. Thus, this paper aims to review the role of HERVs in urologic neoplasms and analyze the feasibility of utilizing HERVs in immunotherapies for urologic neoplasm patients.

## Endogenous retroviruses

Similar to exogenous retroviruses, HERVs possess four core structural genes (env, gag, pol, and pro), flanked at both ends by long terminal repeats (LTRs) harboring regulatory functions [1]. The gag gene encodes the major structural polyprotein Gag, which is processed by host proteases into capsid and nucleocapsid proteins. The env gene encodes envelope (Env) glycoproteins responsible for receptor recognition, binding, and membrane fusion, comprising surface (SU) and transmembrane subunits. The pol gene encodes enzymes crucial for viral replication, including reverse transcriptase (RT) and integrase, facilitating DNA synthesis and integration into the host genome. LTRs contain essential *cis*-acting regulatory elements, such as promoters and enhancers [17–19]. HERVs are phylogenetically classified into three major classes (I, II, III) based on pol gene sequence similarity. Further subdivision into distinct groups relies on the complementarity of their primer binding site sequences to specific transfer RNAs [1, 20, 21].

### Regulatory mechanism of HERVs

**Fig. 2:**
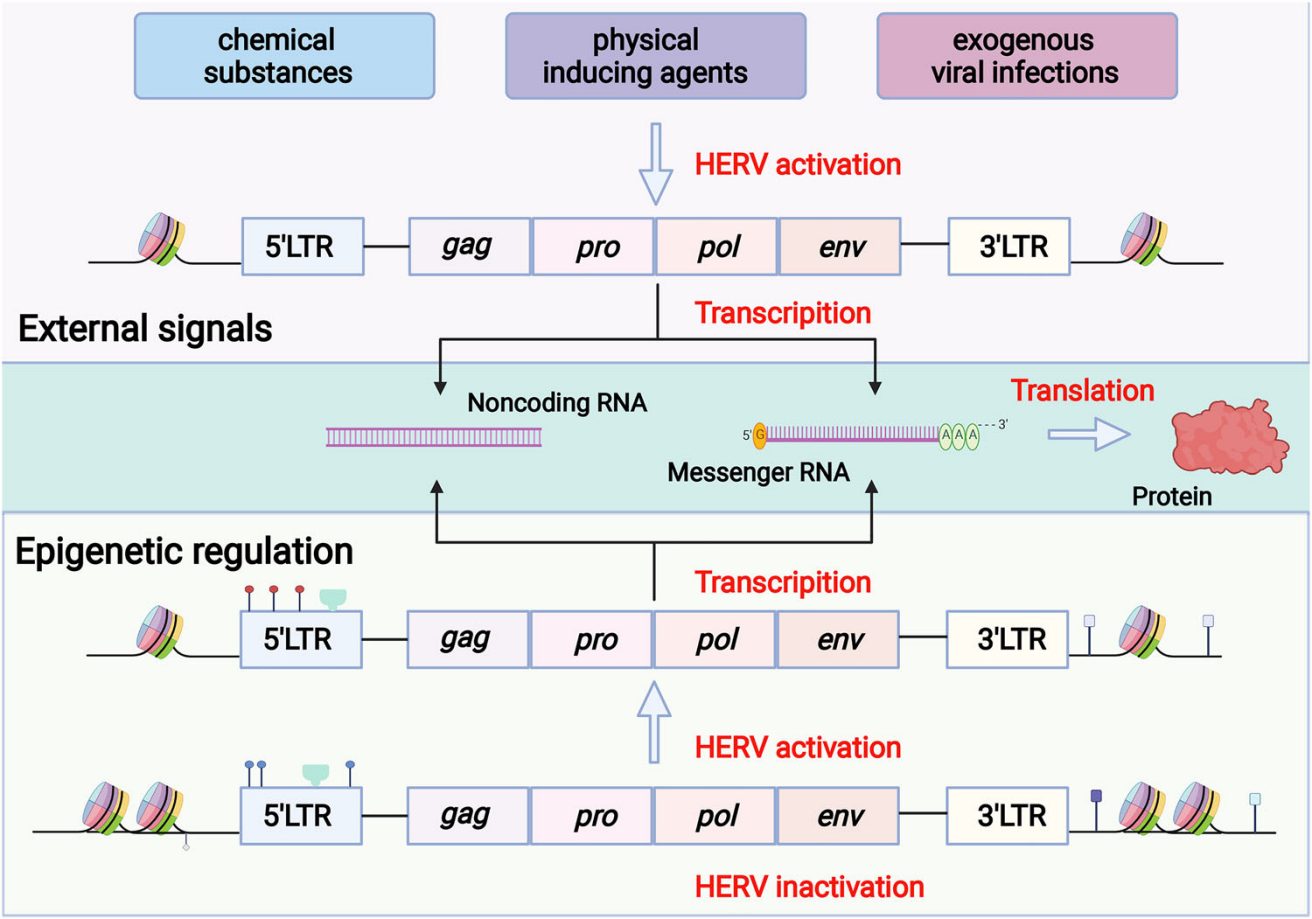
The activation of HERV is influenced by environmental factors and epigenetic modifications. External environmental factors, such as chemical substances, physical inducing agents, and exogenous viral infection, can upregulate the expression of HERVs; epigenetic regulation by DNA methylation and histone modification can also upregulate the expression of HERVs (Created with BioRender.com).

Although most HERVs have lost transcriptional activity over millions of years of evolution, consensus indicates they can be reactivated by external stimuli and epigenetic dysregulation [4, 20, 22, 23] (Fig. 2). Substantial evidence shows HERVs expression can be upregulated by environmental factors, including chemical compounds, physical agents, and exogenous viral infections.

For instance, exogenous brain injury activates HERVs expression [24], γ-irradiation upregulates HERV-R [25], and ultraviolet C irradiation induces HERV-K expression [26]. Copper ions exhibit a dual role in melanoma, potentially inducing or inhibiting HERV activation depending on concentration [27]. Interferon-γ stimulation promotes HERV expression in tumor cells [28], while silver nanoparticles induce HERV-W expression in a size-dependent manner [29]. However, the precise molecular mechanisms underlying HERV activation by these physical and chemical factors remain poorly defined.

Exogenous viral infections are potent inducers of HERV expression. In multiple sclerosis (MS), Epstein-Barr virus triggers HERV-K transactivation in lymphoblastoid cell lines [30] and may induce early HERV-W expression via activation of human herpesvirus-6A in the central nervous system, contributing to MS pathogenesis [31, 32]. Elevated HERV expression is observed in HIV-1-infected individuals [33, 34]. Specifically, the HIV-1 Tat protein activates HERV-K transcription through the NF-κB and NF-AT pathways [35–37]. Latent proteins LANA and vFLIP encoded by Kaposi’s sarcoma-associated herpesvirus modulate MAPK/NF-κB signaling and cytokine production, mediating HERV-K activation implicated in Kaposi’s sarcoma (KS) development [34, 38, 39]. Human cytomegalovirus (HCMV) infection increases HERV expression in various cells [40]; clinically, HCMV infection associates with elevated HERV-K and HERV-W expression in renal transplant recipients, although the specific mechanisms are undefined [41, 42].

Epigenetic regulation constitutes the primary host mechanism controlling HERV expression levels [43], primarily through DNA methylation and histone modifications [44]. Regulatory proteins and small RNAs also contribute to HERV activation [45, 46].

Accumulating evidence suggests that DNA hypomethylation (demethylation) derepresses HERVs, leading to aberrant expression. Genome-wide studies reveal lower HERV DNA methylation levels in human embryonic stem cells compared to differentiated cells, like fetal lung fibroblasts, correlating with increased transcriptional activity downstream of HERV LTRs [47]. Correspondingly, DNA methylation is recognized as a key mechanism for silencing HERVs and other repetitive elements [48–50]. Specifically, in melanoma, LTR hypomethylation correlates with elevated HERV-K expression and a more malignant phenotype [51]. In placental and choriocarcinoma cells, HERV-W promoter methylation inversely correlates with its mRNA and protein expression [52]. Hypomethylation contributes to activated HERV-H expression in head and neck tumors compared to normal tissues [53]. Furthermore, varying degrees of HERV hypomethylation are reported in urothelial, testicular, ovarian, and other malignancies [51, 54, 55], collectively implicating DNA hypomethylation as a major driver of HERV derepression.

Histone acetylation and methylation also significantly impact HERV expression, exhibiting a more complex relationship than DNA methylation [46]. Histone acetylation generally promotes open chromatin and transcriptional activation of associated HERVs [19, 56]. For example, several investigators have induced HERV-H expression in cancer tissue using histone acetylation inhibitors [57]. Furthermore, inhibition of histone deacetylases can interfere with HERV-Fc1 silencing in a variety of human cells, which means that HERV activation and expression are promoted by histone acetylation [58]. The impact of histone methylation on HERVs is more complex than that of histone acetylation, and different methylation types have different effects on gene activity [44, 46, 59]. In B lymphocytes deficient in the histone methyltransferase SETDB1, ERVs are activated in a lineage-specific manner due to the difference in transcription factors that target proviral regulatory elements [60]. However, the silencing mechanism of LTRs could be related to the evolutionary age of LTRs. Unlike early evolutionary LTRs that are mainly silenced by DNA methylation, intermediate-age LTRs may be more sensitive to histone H3 lysine 9 (H3K9) methylation silencing; researchers have shown that the histone methyltransferases EHMT2, SETDB1, and SUV39H1 are involved in LTRs silencing in humans [61]. Of concern, there may be reciprocal links between histone acetylation and histone methylation, and one lysine acetyltransferase, Tip60, can promote histone H3K9 trimethylation by positively regulating the histone methyltransferases SUV39H1 and SETDB1, thereby inhibiting HERV expression in colorectal cancer cells to suppress malignancy development [62]. In summary, studies on the regulatory role of histone modifications in HERVs are still quite limited.

**Fig. 3:**
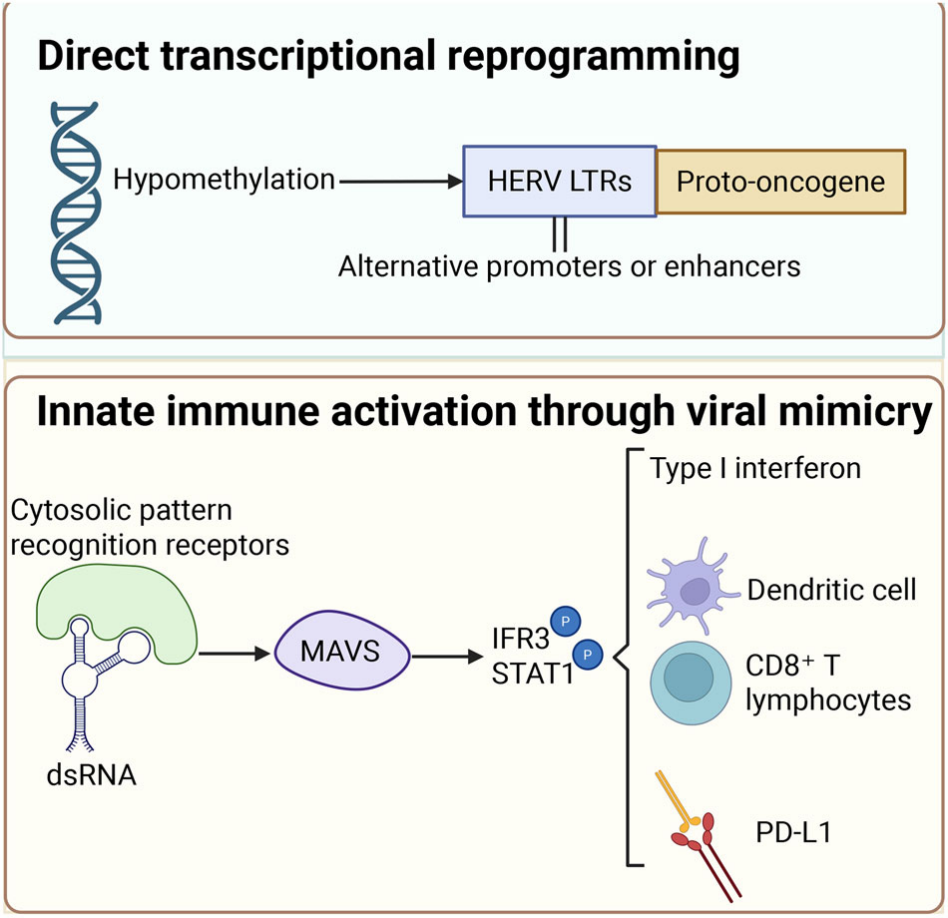
Mechanistic link between HERV epigenetic derepression and oncogenesis. DNA hypomethylation silencing reactivates LTR elements, functioning as alternative promoters/enhancers for proto-oncogenes and driving oncogenic transcriptional programs. In parallel, derepressed HERV transcripts form double-stranded RNAs that activate MAVS–IRF3/STAT1 signaling, inducing type I interferon responses and immune cell recruitment, but also upregulating PD-L1 to establish compensatory immunosuppression (Created with BioRender.com).

Collectively, epigenetic derepression initiates oncogenic cascades through direct transcriptional reprogramming and innate immune activation (Fig. 3). Direct transcriptional reprogramming: Genome-wide DNA hypomethylation reactivates LTR elements that function as alternative promoters or enhancers for proto-oncogenes. In Hodgkin’s lymphoma, THE1B LTR hypomethylation drives lineage-inappropriate expression of the colony-stimulating factor 1 receptor (CSF1R), which is essential for tumor survival [63]. Acute myeloid leukemia exploits derepressed ERV-derived enhancers—normally silenced by H3K9me3 modifications—to establish oncogenic transcriptional circuits governing apoptosis resistance [64]. This transcriptional hijacking extends to solid tumors, where LTR hypomethylation activates HERV-K expression in melanoma [51] and reactivates placenta-restricted HERV-W loci in testicular carcinomas [54], with parallel hypomethylation patterns documented in head/neck and ovarian malignancies [53, 55].

Innate immune activation through viral mimicry: Hypomethylation-induced HERV transcripts [53–55] or SETDB1/H3K9me3-mediated derepression [59] generate double-stranded RNA (dsRNA) species that engage cytosolic pattern recognition receptors. This activates the mitochondrial antiviral-signaling protein (MAVS) cascade, triggering IRF3-STAT1 phosphorylation and subsequent type I interferon production [28]. While this response recruits dendritic cells and CD8⁺ T lymphocytes-creating an immunogenic microenvironment [28]-it concurrently induces compensatory immunosuppressive checkpoints, including PD-L1 [59]. Therapeutically, WEE1 inhibition exploits this pathway by inducing endogenous retroviral dsRNA to potentiate anti-PD-L1 efficacy [59].

### Potential mechanisms of HERVs-mediated oncogenesis

**Fig. 4:**
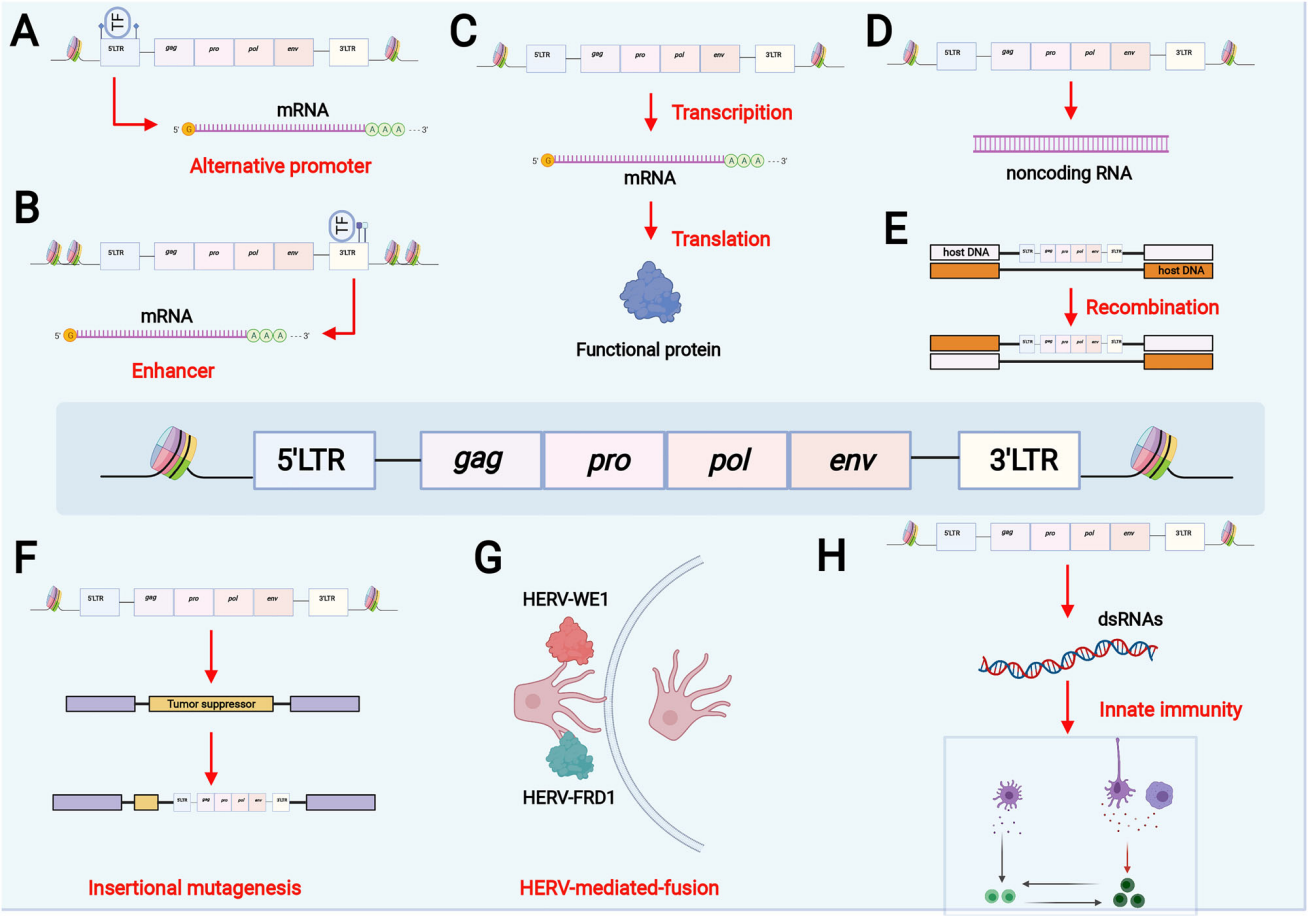
Potential mechanisms of HERV-mediated oncogenesis. HERVs can be involved in the expression of oncogenes as alternative promoters or enhancers of oncogenes. Expression products or ncRNAs of HERVs can also be involved in tumor formation. In addition, HERV-mediated gene recombination and insertion mutations may also lead to the activation of oncogenes or the disruption of oncogenes. Finally, HERVs may also promote tumor formation through cell fusion or by evading immune surveillance (Created with BioRender.com).

The generation of malignancies is an extremely complex process that results from the activation of oncogenes or the inactivation of tumor suppressor genes due to a series of genetic, epigenetic, or environmental factors [65–67]. Mechanistically, these processes originate from epigenetic HERV derepression (Section “Regulatory mechanism of HERVs”), which drives transcriptional hijacking of oncogenes and dsRNA-dependent immune remodeling. Available evidence suggests that HERVs also play a role in malignancy generation through various mechanisms (Fig. 4).

HERVs can activate the expression of oncogenes as alternative promoters [15, 68–70]. For example, Hodgkin’s lymphoma cells can aberrantly express CSF-1R and are sensitive to CSF-1R inhibitor therapy; thus, the CSF-1R pathway could be a potential target for the treatment of Hodgkin’s lymphoma. However, CSF-1R transcription in Hodgkin’s lymphoma does not originate from its normal promoter but from an LTR element of the MaLR THE1B family upstream of its normal promoter [63], and the Hodgkin-Reed-Sternberg gene transcribed from activated LTRs plays a role in determining the phenotype of classical Hodgkin lymphoma [71]. HERVs can also enhance the expression of relevant oncogenes. In acute myeloid leukemia, the chromatin profiles of ERVs are thought to include enhancers, and ERVs are thought to be associated with disease phenotypes and cancer progression [64]. The transcribed and translated HERV protein products influence tumor formation by affecting signaling pathways and inducing tumor immune escape [20, 72, 73]. Noncoding RNAs (ncRNAs) from HERVs have the ability to affect genome function [46]. Studies have shown that ncRNAs from HERVs are highly expressed in human triple-negative breast cancer (TNBC), and these ncRNAs bind to the metastasis suppressor ZMYND8, thereby promoting the proliferation and invasion of TNBC and leading to a poor prognosis [74]. In addition, many highly conserved endogenous retroviral-associated adenocarcinoma RNAs are specifically activated in adenocarcinomas and are associated with poor survival rates [75]. Phylogenetic and sequence analyses of HERV-K have shown that at least 16% of the elements undergo significant rearrangements [76], which may contribute to copy number variations generated by nonallelic homologous recombination mediated by HERVs [77]. HERV-mediated insertions may lead to the activation of oncogenes or the disruption of tumor suppressor genes [46, 78], but this mechanism still needs to be further explored and validated. One of the important functions of HERVs is trophoblast cell fusion mediated by synctin-1, which is encoded by HERV-W, and the syncyin-2 protein, which is encoded by HERV-FDR [79, 80] and is essential for normal placental development [81]. In melanoma, the HERV-K-encoded protein can mediate the fusion of melanoma cells, which may give rise to multinucleated cells and cause genetic evolution [82]. HERVs paradoxically shape immunogenicity through epigenetic dsRNA release. While MAVS-IRF3-STAT1 signaling recruits T cells [28], compensatory PD-L1 induction facilitates evasion [59]. Notably, certain HERV proteins (e.g., syncytin-2) directly suppress immunity [83]. In addition, there are many other HERV ENV proteins with immunosuppressive effects [19] whose immunosuppressive mechanisms are unknown, and some studies suggest that they may indirectly reduce the activation and proliferation of T cells by regulating the activity of dendritic cells [84].

## The biological role of endogenous retroviruses in urologic neoplasms

ERVs/HERVs demonstrate multifaceted roles in urologic oncogenesis, contributing to tumor development through epigenetic dysregulation, immune modulation, and viral mimicry across major malignancies. As detailed in subsequent sections and synthesized in Table 1, these biological mechanisms manifest distinctly in prostate cancer (androgen-responsive HERV-K activation), renal cell carcinoma (RCC; HIF-driven ERV immunogenicity), bladder cancer (HERV-derived miRNA silencing), and testicular germ cell tumors (hypomethylation-associated HERV expression). Table 1 provides a systematic overview of these pathogenetic mechanisms and their clinical implications.

**Table 1:** The biological roles and mechanisms of endogenous retrovirus in urologic neoplasms.

Cancer type	Key target	Biological roles	Mechanism	References
**Prostate cancer**	HERV-K (HML-2)	Tumor promotion; Immune modulation	Reduced LTR methylation increases expression; Androgen-responsive LTR elements; ERG-mediated transcriptional activation	85, 89–93
	HERV-E Env	Tumor-specific antigen	Selective expression in tumor tissues	88
	HERV-K Gag	Immune activation	Demethylation-dependent expression induces antigen-specific T-cell response	92
	HERV-K locus	Genomic instability	Ionizing radiation induces recombination mutations	94
**Renal cell carcinoma**	HERV-E Env	Immunogenicity; T-cell activation	HLA-A*02:01-restricted peptide presentation; Tumor-infiltrating lymphocyte recognition	102
	ERV-derived peptides	Antitumor immunity	HIF-driven ERV expression enables HLA presentation triggering CD8⁺ T-cell response	104
	POU5F1-HIF heterodimer	Malignant progression	HERV LTR reactivation dysregulates stemness pathways	107, 108
	HERVERI superfamily	Epigenetic dysregulation	PBRM1 loss induces HIF-dependent derepression	111, 112
	CT-RCC HERV-E antigen	TCR therapy target	Engineered TCRs selectively kill ccRCC cells	120
**Bladder cancer**	Syncytin-1 (HERV-W Env)	Oncoprotein; Tumor invasion	3′-LTR mutation (142T>C) enables c-Myb binding causing transcriptional activation	122, 123
	miR-4454 (HERV-H-derived)	Tumor suppressor silencing	Targets *DNAJB4* and *SASH1* increasing proliferation/invasion in NMIBC	124
	HERV-EC1	Metabolic reprogramming	Antagonizes *PLA2G4A* reducing cPLA2 promoting carcinogenesis	125
**Testicular GCTs**	HERV-K	Biomarker; Immune evasion	Serum antibody detection; Signal peptide mediates immune escape	131–136
	Syncytin-1 (HERV-W Env)	Cell fusion; Tumor specificity	U3 hypomethylation activates promoter	54, 138
	HERV transcripts	Inflammatory microenvironment	DNA hypomethylation increases expression, inducing IFN-α signaling and CD8⁺ infiltration	137

*HIF* hypoxia-inducible factor, *LTR* long terminal repeat, *NMIBC* non-muscle-invasive bladder cancer, *GCTs* germ cell tumors, *TCR* T-cell receptor, *ccRCC* clear cell renal cell carcinoma.

### Prostate cancer

The expression of HERVs is linked to the development of prostate cancer [85], and in a comprehensive study exploring the site-specificity of HERVs expression in prostate, breast, and colon cancers, researchers identified active HERVs elements that exhibit distinct expression patterns in these malignancies, in addition to the differentially expressed host genes involved in the demethylation and antiviral response pathways, which may be relevant to cancer development [86]. A total of 155 HERVs are differentially expressed in all three cancers, and the identification of this HERVs expression pattern provided the basis for further subsequent studies on the function of HERVs in cancer and potential biomarkers and therapeutic targets for cancers [86]. One of the more active and well-studied transcripts is that of HERV-K and its subgroup HML-2, and in a study by Agoni et al., they detected transcription products of the HERV-K (HML-2) coding chain in the LNCaP, DU145, PC3, and VCaP prostate cancer cell lines. In further identifying the HERV-K locus, they found that the sequences within the viral genome were mostly smaller fragments, but the transcribed sequences of their LTRs were relatively intact, and either of the two strands in their locus could be transcribed [87]. In addition to HERV-K, the HERV-E env gene is specifically expressed in prostate cancer tissues, and it is hardly expressed in healthy prostate tissues [88].

The expression of HERVs in prostate cancer may be associated with host factors and environmental factors. A study by Goering et al. found that in prostate cancer, reduced DNA methylation of LTRs led to a significant increase in HERV-K_22q11.23 provirus expression in neoplasms, but HERVK17 expression was reduced and independent of the degree of methylation of LTRs [89]. Notably, the transcription of these HERVs was largely restricted to androgen-responsive prostate cancer cell lines, and their LTRs contained steroid hormone response elements associated with androgen expression and regulation; thus, the differential expression of different HERVs may be related to androgen receptors and androgen responsiveness. Two possible explanations for this relationship are: 1. the fact that LTRs are located near the androgen binding site and associated cofactors, and 2. the highly expressed transcription factor ERG is present in tumor tissues with high expression of HERV-K_22q11.23 [89, 90]; high expression of ERG has been shown to affect androgen responsiveness of the locus [91]. A study by Reis et al. identified a HERV-K GAG protein that was highly expressed on chromosome 22q11.23 in prostate cancer cells and produced a specific immune response in advanced prostate cancer; subsequent studies found that high expression of this protein correlated with the demethylation of LTRs and sensitivity to androgen response [92]. Goering et al. suggested that the specific expression of HML-2 in malignancies and tissues is more likely to be determined by the chromatin environment, based on factors such as DNA demethylation, rather than by specific transcription factors [93]. In addition to the possible host factors mentioned above, environmental factors can also affect the expression of HERVs. Ionizing radiation was found to affect the transcription levels of HERV-K in prostate cancer cells, but such effects are transient, usually peaking after 24 h of a single 2.5–20 Gy gamma irradiation and returning to basal levels within 72 h [94]. This, in turn, may have a series of knock-on effects, as follows: first, since the HERV-K protein is recognized by the immune system [95, 96], ionizing radiation may enhance the body’s antitumor immune response; second, as previously mentioned, HERV-K overexpression may promote the development of prostate cancer [97]; and third, ionizing radiation may lead to recombination mutations in HERV-K motifs in the genome [94]. In addition, Merrick et al. found that under long-term arsenic exposure, HERVs undergo derepression and increased expression due to epigenetic alterations and genetic recombination; this may also be a mechanism by which arsenic exposure can lead to the development and spread of prostate cancer [98, 99].

Rubenstein et al. hypothesized that an interferon-based antiviral defense system exists in prostate cancer and that changes to this antiviral system based on the overexpression of HERVs may extensively impact lymphocyte expression and immune regulation in prostate cancer [100]. Consistent with their findings, Molinaro et al. found that as 2’,5’ oligoadenosine synthase (OAS) can form 2’,5’-linked oligoadenosine (2–5A) upon the activation of viral dsRNA, under the induction of interferon, RNase L can exert antiviral and antitumor activity by binding to the allosteric effectors 5’-phosphorylated 2–5A; furthermore, RNase L mutations are risk factors for prostate cancer. Further studies revealed that HERV env RNAs bind to and activate OASs in the prostate cancer cell line PC3; thus, overexpression of HERV env RNAs in prostate cancer may play a contributory role in the induction of antiviral and antitumor activity [101].

### Renal cell carcinoma

Clear cell RCC (ccRCC) represents the most common histologic subtype of RCC, accounting for approximately 70% of cases. A seminal study by Cherkasova et al. demonstrated selective expression of a transcript encoding the HERV-E envelope gene in the majority of ccRCC tissues, contrasting with its absence in normal kidney tissue. Critically, they identified HLA-A*0201-restricted peptides derived from this HERV-E envelope transcript capable of stimulating CD8 + T cells, providing initial evidence for its immunogenicity [102]. Further supporting this, a recent comprehensive proteogenomic analysis of the HLA class I immunopeptidome in RCC tissues revealed the presentation of not only canonical peptides but also peptides derived from hERVs. This study identified tumor-associated hERV antigens, with one demonstrating immunogenicity recognized by host tumor-infiltrating lymphocytes (TILs). Remarkably, stimulation with this hERV antigen also induced reactive CD8 + T cells from healthy donor PBMCs, highlighting the existence of anti-tumor CD8 + T cell surveillance targeting hERV-derived antigens in RCC and their translational potential [103]. Mechanistically extending these findings, a pivotal study specifically investigated the role of hypoxia-inducible factor (HIF) transcription factors in regulating ERV expression in ccRCC. The authors demonstrated that HIF directly upregulates multiple ERVs in ccRCC. Crucially, some ERV-derived peptides were shown to be presented on HLA molecules and capable of eliciting T-cell responses. This work holds significant implications for exploiting ERVs as targets for tumor immunotherapy, as it suggests HIF stabilizers could potentially induce ERV expression in non-ccRCC tumors. Importantly, it provides a mechanistic explanation for the notable immunogenicity of ccRCC despite its relatively low tumor mutation burden [104]. Additional studies corroborate the association between aberrant HERV expression and ccRCC pathogenesis [105]. Mechanistically, the activation of HERVs is often driven by their LTRs [106]. Dormant promoters within HERV LTRs can be reactivated; notably, HIF binding near the stem cell transcription factor POU5F1 (OCT4) can generate a novel POU5F1 heterodimer. This heterodimer correlates with advanced malignancy stage and poor overall prognosis in ccRCC patients [107, 108].

PBRM1 inactivation is the second most frequent mutation in ccRCC and is intricately linked to tumor development and variable responses to immune checkpoint blockade (ICB) [109, 110]. Recent findings indicate that PBRM1 loss negatively regulates HERVs in a HIF1α- and HIF2α-dependent manner, particularly leading to increased expression of the HERVERI superfamily [111, 112]. Intriguingly, the tumor microenvironment (TME) significantly influences immune-related factors in RCC. Research on the immune checkpoint HHLA2 revealed its expression in primary ccRCC tumors ex vivo, which diminished during in vitro culture. While ccRCC cell lines (A498, 786-O) lacked HHLA2 expression in vitro, HHLA2 was induced upon their engraftment as xenografts in immunodeficient mice, underscoring the TME’s role in its regulation. Furthermore, specific cytokines (IL-10, BMP4, and to a lesser extent IL-1β, IL-6) were found to modestly enhance HHLA2 expression in monocytes and dendritic cells, though they failed to induce it in ccRCC cell lines in vitro [113]. However, the relationship between PBRM1 status and prognosis appears complex, as some reports suggest ccRCC patients with PBRM1 inactivation may have a better prognosis compared to those with other mutations- seemingly contradicting the association between elevated LTR-driven HERV expression and poor outcomes [110, 114, 115]. This paradox can be resolved by TME heterogeneity: (1) HERV-E overexpression driven by PBRM1 loss promotes immune escape via upregulation of T-cell exhaustion markers (TIM-3, LAG-3) [105, 116]; (2) In inflamed TMEs, PBRM1 deficiency induces cytosolic DNA sensing through defective G2/M checkpoint control, enhancing response to immune checkpoint inhibitors (ICIs) [117]; (3) Hypoxia level and immune cell density determine whether HERVs exert pro-tumor or immunogenic effects [118]. Earlier work by Cherkasova et al. proposed that Von Hippel-Lindau inactivation underlies selective HERV-E expression in ccRCC, showing linear correlation with HIF-2α levels; paradoxically, they also observed hypermethylation of LTRs in ccRCC [119], suggesting that expression levels may involve a complex interplay beyond simple demethylation.

Most significantly, recent advances have propelled HERV-E from a biomarker to a therapeutic target in ccRCC. A landmark study described the cloning and characterization of a novel T cell receptor (TCR) specific for a HERV-E-derived antigen (CT-RCC HERV-E), which is uniquely expressed in ccRCC. Engineered T cells expressing this HERV-E TCR selectively recognized and killed ccRCC tumor cells both in vitro and in mouse xenograft models. This represents the first demonstration that ccRCC cells can be effectively targeted by TCR-engineered T cells directed against a HERV-derived antigen. The study also established a GMP-compliant method for large-scale production of these HERV-E-reactive T cells, providing a robust preclinical foundation for evaluating adoptive T cell therapy with HERV-E TCR-transduced T cells in patients with advanced ccRCC [120].

### Bladder cancer

On a global scale, bladder cancer ranks as the tenth most prevalent malignancy, with approximately 573,000 new cases reported in 2020 [5]. Over 90% of patients are diagnosed with urothelial carcinoma [121]. Accumulating evidence implicate HERVs in bladder carcinogenesis through diverse mechanisms.

A significant finding involves HERV-W Env (Syncytin-1), which exhibits overexpression in 75.6% of bladder urothelial carcinomas (BUC) and demonstrates potential oncogenic properties. This dysregulation is mechanistically linked to specific 3’-LTR mutations, notably the 142T>C substitution. The mutated 3’-LTR serves as a binding site for transcription factor c-Myb, resulting in transactivation of the Syncytin-1 promoter and consequent protein overexpression [122, 123]. In addition to protein-coding effects, aberrant HERV activity can manifest through ncRNAs. A genome-wide analysis identified 29 microRNAs (miRNAs) originating from HERV sequences. Critically, miR-4454, derived specifically from HERV-H, was found upregulated in non-muscle invasive bladder cancer (NMIBC) cells. Functional characterization revealed that miR-4454 directly targets and suppresses the tumor suppressor genes *DNAJB4* and *SASH1*, thereby promoting NMIBC progression [124]. Another study found that HERV-EC1, a provirus of HERV-E4-1, antagonizes the transcription of PLA2G4A, thereby inhibiting the expression of its encoded cytoplasmic phospholipase A2 (CPLA2), which in turn promotes uroepithelial carcinogenesis [125]. Furthermore, smoking promotes the transcription of HERVs in normal urinary epithelium [126], and as smoking is the most important trigger for bladder carcinogenesis [127], increased expression of HERVs may be associated with the development of bladder cancer.

### Testicular germ cell tumors

Testicular cancer is the most common malignancy in young men, and its incidence rate is increasing [128], with the vast majority of testicular cancer tumors being germ cell tumors (GCTs) and a few non-germ cell tumors [129]. Testicular germ cell tumors (TGCTs) are the most common GCTs, and TGCTs account for approximately 95% of all GCTs. Approximately 55–60% of GCTs are seminomas, and the rest are non-seminomas [130].

Several studies have shown that GCT is closely associated with HERV-K expression [131, 132] and that HERV-K protein antibodies have the potential to be markers of GCT [131, 133–135]. Ruggieri and colleagues found that HERV-K encoded a stable signal peptide, and this signal peptide may be associated with GCT malignancy formation and immune escape [136]. Several additional studies have suggested that HERVs may be related to the development of seminomas and DNA hypomethylation. Compared to non-seminomas, DNA hypomethylation and CD8+ lymphocyte infiltration are increased in seminomas, and such DNA hypomethylation is accompanied by a marked increase in the expression of HERVs; meanwhile, RNA in situ hybridization showed that the expression of such HERVs is restricted to cancer cells [137]. This study also showed increased expression of interferon alpha in seminomas compared to that in non-seminomas, and this increased interferon response was mediated by cancer cells [137]. Therefore, the authors speculated that DNA hypomethylation in seminomas may be associated with elevated HERV expression, CD8+ lymphocyte infiltration and interferon α signaling, which may also explain the characteristic lymphocyte infiltration in seminomas [137]. A study conducted by Benešová et al. found that in non-seminomas, DNA hypomethylation was also associated with syncytin-1 derepression, and the resulting syncytin-1 splice RNA could serve as a marker for non-seminomas [138]. In a study using a custom-made HERV high-density microarray, the investigators found that six HERV-W motifs are highly expressed in testicular cancer cells, including the syncytin-1 transcript; they also showed that DNA hypomethylation of the U3 region promoter is a prerequisite for syncytin-1 activation [54].

## The clinical significance of hervs in urologic neoplasms

HERVs demonstrate significant clinical utility as diagnostic biomarkers and therapeutic targets across major urologic malignancies. As synthesized in Table 2, HERVs underpin emerging clinical applications including prostate cancer diagnostic refinement, predictive stratification for ICIs in RCC, and prognostic assessment in bladder cancer. Table 2 provides a comprehensive summary of these clinically actionable dimensions of HERV biology in urologic oncology.

**Table 2:** The clinical applications of HERVs in urologic malignancies.

Cancer type	Clinical application	Specific target/approach	Key findings	References
**Prostate cancer**	Diagnosis	HERV-K GAG RNA/protein	Elevated in 85.2% tumors vs. 5.6% benign tissues; plasma antibodies detected	142–144
	Diagnosis	Prostate-specific HERV probes	44 HERV probes identified via microarray; requires clinical validation	141
	Diagnosis	PSA + HERV-K assay	Enhances detection sensitivity in older men/smokers	142
	Therapy	RT inhibitors (e.g., abacavir)	Inhibits tumor growth, S-phase delay, reduces PC3/LNCaP invasion	145, 146
	Therapy	HERV-K GAG vaccines	Triggers B-cell autoantibodies in advanced disease; induces CD8+/CD4+ response	92, 147
**Renal cell carcinoma**	Prognosis	ERV-based stratification (E4421/E1659)	Predicts ICI response in metastatic ccRCC; outperforms transcriptomic signatures	151
	Prognosis	HHLA2	Elevated expression correlates with advanced clinicopathological features	152
	Therapy	Vaccines (MVA-HKenv/MVA-HKcon)	Reduces lung metastases in kidney cancer models	153, 154
	Therapy	Epigenetic-ICI combination	DNMTis enhance ICI efficacy via interferon signaling	46, 48, 160–162
	Therapy	ACT/mAb targeting PD-1	High PD-1⁺/low TIM-3⁻/LAG-3⁻ TILs predict nivolumab response in metastatic ccRCC	159
**Bladder cancer**	Diagnosis	miR-4454 (HERV-H-derived)	Upregulated in NMIBC; early detection biomarker	124
	Prognosis	HHLA2	Independent predictor of tumor size/stage/grade/LN metastasis	163
	Diagnosis	UCA1 (HERV-H-associated)	High sensitivity/specificity for bladder cancer detection	164

*ACT* adoptive cell therapy, *DNMTis* DNA methyltransferase inhibitors, *ICI* immune checkpoint inhibitor, *LN* lymph node, *NMIBC* non-muscle-invasive bladder cancer, *RT* reverse transcriptase, *TILs* tumor-infiltrating lymphocytes.

### Prostate cancer

The high expression of HERVs in prostate cancer suggests that HERVs could become specific biological markers and therapeutic targets for prostate cancer. Although prostate-specific antigen (PSA) has now become the main screening tool for prostate malignancies clinically, PSA has low specificity and sensitivity [139, 140]. Previous researchers designed a high-density Affymetrix format microarray that could target the HERV-W, HERV-H, HERV-E 4.1, HERV-FRD, HERV-K HML-2, and HERV-K HML-5 families of 2690 different proviruses and 2883 individual LTRs; 44 prostate-specific HERV probes were identified, but further clinical studies are needed to validate the sensitivity and specificity of these biomarkers [141]. A study by Wallace et al. found significantly higher levels of HERV-K GAG transcripts in peripheral blood nuclei in prostate cancer patients than in healthy men; they also found that this high level was associated with elevated plasma interferon-gamma concentrations, especially in older men and smokers with increased sensitivity. Thus, combining the PSA assay with the HERV-K assay may enhance the efficiency of prostate cancer diagnosis, especially in older men and smokers [142]. A study by Rezaei et al. further confirmed that HERV-K GAG RNA levels were significantly elevated in 85.2% of prostate cancer tissues. Meanwhile, expression of the HERV-K GAG protein was found in 66.7% (12/18) of malignant tissues, while it was found in only 5.6% (1/18) of benign tissues. Therefore, high expression of HERV-K GAG RNA and protein may be a sensitive and specific biological marker of prostate cancer, and this could significantly contribute to clinical diagnosis [143]. Manca et al. found a significant humoral plasma response to the HERV-K and HERV-H antibodies in prostate cancer patients; this finding provides additional evidence of the association between HERV-K and prostate cancer and suggests that HERV-H may also be involved in the development of prostate cancer [144].

In regard to treatment, a study by Landriscina et al. was conducted based on the premise that RT activity is associated with the treatment response of cancer cells with low levels of differentiation. The authors showed that non-nucleoside RT inhibitors, which are used for HIV treatment, had potent cytostatic and differentiation activity in several models of cancer, including those of prostate cancer. Therefore, the authors speculated that this activity might be associated with limiting endogenous RT activity and that RT inhibitors could enhance therapeutic efficacy in hormone-insensitive prostate cancer under specific circumstances; however, this conjecture still needs to be confirmed by further clinical trials [145]. Carlini et al. found that abacavir (a nucleoside reverse transcription inhibitor) not only significantly inhibited prostate cancer cell growth, thus causing a delay in S-phase progression in the cell cycle, but also reduced the invasion and migration of PC3 and LNCaP cells. The mechanism underlying this phenomenon is unclear, and the authors speculated that the mechanism may be related to the upregulation of LINE-1 expression in prostate cancer cells [146]. Meanwhile, as previously mentioned, GAG may also serve as a target for immunotherapy due to the high expression of HERV-K GAG and its immunogenicity, especially in advanced prostate cancer, where high HERV-K GAG expression triggers autoantibody production by B cells [92]. The following two options have been proposed regarding vaccines against HERVs: 1. the generation of a mixed CD8 + /CD4+ response in the tumor by the vaccine and treatment by activated T cells; and 2. the use of a vaccine against GAG in men with elevated PSA and inert or gray area cancers [147]. However, both options require further clinical trials to validate their effectiveness. Potential off-target effects require careful evaluation, particularly concerning cross-reactivity with physiologically essential HERV-derived proteins such as placental syncytin-1 (HERV-W Env), which mediates critical trophoblast fusion functions during embryonic development [148, 149]. Encouragingly, recent advances demonstrate tumor-selective targeting solutions: TCR-engineered T cells directed against tumor-specific HERV-E epitopes (e.g., CT-RCC antigen in RCC) achieve malignant cell elimination without damaging normal renal tissue [120], while monoclonal antibodies against HERV-K exhibit high-affinity binding to breast cancer cells with minimal reactivity to normal breast epithelia [150]. These findings support the feasibility of developing precision immunotherapies that mitigate off-target risks.

### Renal cell carcinoma

The abnormal expression of HERVs in ccRCC indicates that HERVs could serve as diagnostic and prognostic markers for ccRCC. Recently, a novel ERV-based stratification system was developed to predict the efficacy of ICIs therapy in patients with metastatic ccRCC. By analyzing ERV expression in two cohorts of metastatic ccRCC patients (*n* = 181 receiving nivolumab in CheckMate trials; *n* = 48 receiving ipilimumab-nivolumab in BIONIKK trial), two specific ERVs (E4421_chr17 and E1659_chr4) with opposite prognostic impacts were identified. A four-tiered risk stratification model based on these ERVs significantly correlated with ICIs outcomes and outperformed traditional transcriptomic signatures in predicting ICIs efficacy. Integrating ERV expression with epigenetic markers further refined the predictive accuracy, highlighting the potential for personalized ICIs therapy optimization in metastatic ccRCC [151]. The expression of HERV-H long terminal repeat-associated protein 2 (HHLA2) is elevated in ccRCC and associated with various clinical and pathological features. Comprehensive microarray analysis shows that the target of HHLA2 plays a role in biological processes involved in the immune response [152]. Therefore, HHLA2 may be a potential prognostic marker for ccRCC.

In terms of immunotherapy, HERVs serve as targets for tumor vaccines, as well as for adoptive cell therapy (ACT) and monoclonal antibody (mAb) therapy, and combination therapy with epigenetic therapy and ICIs is possible [108]. In terms of tumor vaccines, some researchers have constructed a recombinant vaccinia virus (MVA-HKenv) that expresses the HERV-K envelope glycoprotein and validated its effects in animal models. In a mouse kidney cancer model, overexpression of HERV-K ENV promoted the formation of lung metastases, whereas a single inoculation with MVA-HKenv significantly reduced lung metastases in mice [153]. In a follow-up experiment by the same investigators, they constructed a recombinant vaccinia virus that expressed the HERV-K Gag protein (MVA-HKcon) and again showed promising results in a mouse model [154]. However, the efficacy and safety validation of these strategies in humans is lacking. ACT and mAb treatment have been validated for their efficacy and safety in other diseases, such as melanoma [155], breast cancer [150, 156], and MS [157, 158], and recent studies have found that CD8+ tumor-infiltrating cells express PD-1 (programmed cell death protein 1) at high levels but not TIM-3 (T cell immunoglobulin domain and mucin domain-3), LAG-3 (lymphocyte activation gene-3), and ERVE4; thus, patients with metastatic ccRCC should have a better response to nivolumab [159]. Nevertheless, in general, the application of ACT and mAb treatment in ccRCC needs to be studied further. In terms of epigenetic therapy and ICI combination therapy, existing studies have shown that abnormal expression of πERVs is associated with immune checkpoint activation in ccRCC, and ERV3-2 expression is associated with ICB efficacy in ccRCC [160]. Meanwhile, DNA methyltransferase inhibitors (DNMTis) can improve the efficacy of ICI through the activation of interferon signaling pathways [46, 48], and their efficacy and safety have been demonstrated in colorectal cancer [161] and ovarian cancer [162], but studies on their efficacy in ccRCC have not been conducted.

### Bladder cancer

A study by Park et al. found that the expression of HERV-H-derived miR-4454 was upregulated in NMIBC and that miR-4454 could serve as a specific biomarker for early NMIBC [124]. HHLA2 was significantly elevated in BUC and significantly correlated with cancer size, stage, grade, and the presence of lymph node metastasis; HHLA2 can be used independently to predict BUC prognosis [163]. The ncRNA gene urothelial carcinoma-associated 1 (UCA1), which is associated with HERV-H, is also a bladder cancer marker with good sensitivity and specificity [164].

## Discussion and future perspectives

HERVs are genomic remnants of ancient exogenous retroviral infections that have been vertically transmitted and fixed within the human germline over millions of years. Although most HERVs sequences have accumulated mutations rendering them incapable of replication or retrotransposition, a significant portion retains the ability to be transcribed, particularly under certain pathological or epigenetically dysregulated conditions. Reactivation of HERVs has been implicated in carcinogenesis through diverse and often tissue-specific mechanisms, including but not limited to transcriptional interference, epigenetic dysregulation, activation of oncogenic signaling pathways, modulation of immune responses, and genomic instability via insertional mutagenesis or recombination events [15].

Several studies have shown a close association between HERVs and urologic neoplasms, including those associated with prostate cancer, bladder cancer, RCC, and testicular cancer. Aberrant expression of HERVs in these malignancies may contribute to their development through different mechanisms [97]. Moreover, monitoring the expression levels of HERVs and their isoforms in human blood may serve as an effective means of early diagnosis or as a prognostic marker for prostate, kidney and bladder cancers [105, 141, 163]. Furthermore, HERVs are potential therapeutic targets due to the immunogenicity of their expression products and their ability to trigger the interferon-based antiviral cascade defense system, which leads to the induction of antitumor immune responses. Previous research has focused on the use of HERVs as targets for prostate cancer or ccRCC vaccines. However, further clinical trials are needed to assess the safety and efficacy of this therapeutic strategy [16]. In summary, HERVs play a critical role in the development of urologic neoplasms through various mechanisms. Understanding the molecular mechanisms underlying the role of HERVs in tumorigenesis may enable us to develop more effective diagnostic and therapeutic strategies for these diseases.

Despite the progress made in recent years, there are still unresolved issues in the study of HERVs and their potential role in urologic neoplasms. For instance, in prostate cancer, although HERV-K activation and the upregulation of its expression in malignancies have been detected, experimental evidence demonstrating the direct pro-carcinogenic effects of HERV-K and its products remains limited [165]. Consequently, some researchers speculate that HERV-K is a result rather than a cause of prostate cancer development. Thus, further experiments are required to clarify the mechanisms underlying the specific activation of HERV-K and its products in prostate cancer [147]. Similarly, in ccRCC, there is conflicting evidence regarding the relationship between the activation of LTRs, HERV overexpression, and tumor staging and prognosis. Some researchers suggest that the activation of LTRs drives the overexpression of HERVs, resulting in the production of a novel POU5F1 isoform that correlates with malignancy stage and poor prognosis. However, other studies indicate that PBRM1 inactivation, which is caused by the second most common mutation in ccRCC, negatively regulates the expression of HERVs in a HIF1α- and HIF2α-dependent manner. Interestingly, ccRCC patients with PBRM1 inactivation have a better prognosis than those with other mutations, contradicting the association between increased HERV expression and poor prognosis in ccRCC. Therefore, further research is required to clarify the complex relationships among HERVs, malignancy staging, and prognosis in ccRCC [107, 108, 111, 112]. Crucially, the dual role of HERVs in PBRM1-deficient ccRCC is dictated by TME heterogeneity. Specifically, under hypoxic conditions, HERV-E overexpression induces T-cell exhaustion markers (TIM-3/LAG-3) that drive immune escape [116]. Concurrently, PBRM1 loss activates the cGAS-STING pathway through DNA damage-induced cytosolic leakage, converting HERV-derived dsRNA into an endogenous adjuvant that synergistically enhances response to immune checkpoint inhibitors within T cell-inflamed tumor niches [117, 118].

These discrepancies underscore the necessity of context-specific analysis and highlight the influence of tumor heterogeneity, microenvironmental factors, and host genetic background on HERV biology. It is plausible that distinct HERV loci exert divergent effects depending on their integration site, regulatory context, and interactions with host transcriptional machinery. As such, a comprehensive understanding of the molecular underpinnings governing the activation and regulation of HERVs in specific tumor contexts is essential. Going forward, critical areas requiring further investigation include the functional characterization of HERV loci through high-resolution CRISPR-based screens and epigenomic profiling, such as ChIP-seq and ATAC-seq, to identify the specific elements that play a functional role in oncogenesis, as opposed to those that are merely transcriptional bystanders. Additionally, exploring the immuno-oncological implications of HERV expression is crucial. Understanding how HERV expression interacts with immune modulation—such as neoantigen presentation, immune checkpoint engagement, and tumor-infiltrating lymphocyte dynamics—could lead to novel combination immunotherapies. Furthermore, large-scale prospective studies are needed to validate HERVs expression as a prognostic or predictive biomarker, integrating it with clinical variables such as tumor stage, grade, therapeutic response, and survival. Therapeutically, given the repressive epigenetic control of HERVs under normal conditions, pharmacological agents, like DNMTis or histone deacetylase inhibitors can induce HERV expression, potentially converting immunologically “cold” tumors into “hot” ones, a strategy known as viral mimicry. Optimizing these approaches for urologic malignancies remains a promising yet underexplored avenue. In conclusion, while HERVs represent a compelling axis in the oncogenic process of urologic neoplasms, their dualistic nature—as both potential drivers and passengers—demands careful dissection. A deeper mechanistic understanding of HERVs activation, regulation, and immunogenic consequences will not only clarify their role in cancer biology but also unlock new diagnostic and therapeutic frontiers in precision oncology.

## Conclusion

This paper provides a comprehensive review of the oncogenic mechanisms of HERVs and recent progress related to HERVs and urologic neoplasms, including the biological mechanisms of HERVs, their potential role in various urologic neoplasms, and the diagnostic and clinical applications of HERVs. Further research on HERVs and their mechanisms of action can promote for their future use in disease diagnosis, targeted therapy, and prognostic assessment.
